# Animal Models of Temporomandibular Joint Osteoarthritis: Classification and Selection

**DOI:** 10.3389/fphys.2022.859517

**Published:** 2022-04-28

**Authors:** Yuqing Zhao, Yanxin An, Libo Zhou, Fan Wu, Gaoyi Wu, Jing Wang, Lei Chen

**Affiliations:** ^1^ Department of Orthodontics, School and Hospital of Stomatology, Cheeloo College of Medicine, Shandong University & Shandong Key Laboratory of Oral Tissue Regeneration & Shandong Engineering Laboratory for Dental Materials and Oral Tissue Regeneration, Jinan, China; ^2^ School of Stomatology, Heilongjiang Key Lab of Oral Biomedicine Materials and Clinical Application & Experimental Center for Stomatology Engineering, Jiamusi University, Jiamusi, China; ^3^ Department of General Surgery, The First Affiliated Hospital of Xi’an Medical University, Xi’an, China; ^4^ School of Basic Medicine, Heilongjiang Key Lab of Oral Biomedicine Materials and Clinical Application & Experimental Center for Stomatology Engineering, Jiamusi University, Jiamusi, China; ^5^ Department of Oral Implants, School of Stomatology, National Clinical Research Center for Oral Diseases & State Key Laboratory of Military Stomatology & Shaanxi Key Laboratory of Stomatology, The Fourth Military Medical University, Xi’an, China; ^6^ Key Laboratory of Shaanxi Province for Craniofacial Precision Medicine Research, College of Stomatology, Xi’an Jiaotong University, Xi’an, China

**Keywords:** temporomandibular joint, osteoarthritis, animal models, induced models, naturally occurring models, genetically modified models

## Abstract

Temporomandibular joint osteoarthritis (TMJOA) is a common degenerative joint disease that can cause severe pain and dysfunction. It has a serious impact on the quality of lives of patients. Since mechanism underlying the pathogenesis of TMJOA is not fully understood, the development of effective tools for early diagnosis and disease-modifying therapies has been hindered. Animal models play a key role in understanding the pathological process of diseases and evaluating new therapeutic interventions. Although some similarities in disease processes between animals and humans are known, no one animal model is sufficient for studying all characteristics of TMJOA, as each model has different translatability to human clinical conditions. For the past 4 decades, TMJOA animal models have been studied by numerous researchers and can be broadly divided into induced, naturally occurring, and genetically modified models. The induced models can be divided into invasive models (intra-articular injection and surgical induction) or non-invasive models (mechanical loading, high-fat diet, and sleep deprivation). Different types of animal models simulate different pathological expressions of TMJOA and have their unique characteristics. Currently, mice, rats, and rabbits are commonly used in the study of TMJOA. This review sought to provide a general description of current experimental models of TMJOA and assist researchers in selecting the most appropriate models for different kinds of research.

## Introduction

Osteoarthritis (OA) is a chronic degenerative condition that often affects the stress-bearing joints, such as the knee, spine, hips, and fingers (Kloppenburg and Berenbaum, 2020). The temporomandibular joint (TMJ), one of the most common and complex joints in the human body, can also be affected by OA. Temporomandibular joint osteoarthritis (TMJOA) is the most common form of arthritis occurring in TMJs due to its high clinical prevalence and consequences on TMJ (Wang et al., 2015). Patients with TMJOA usually have joint pain, swelling and stiffness that leads to activity limitations and even reduced quality of life. Therefore, numerous studies are needed to better understand the development and progression of TMJOA.

The etiology of TMJOA is complex and multifactorial, which is generally considered to be associated with mechanical overloading, abnormal occlusion, trauma, and psychological stress (Tanaka et al., 2008; de Souza et al., 2012). However, the causes of impaired cartilage and subchondral bone of TMJ remain unclear. Currently, the treatments of TMJOA mainly aim to reduce pain, restore TMJ function, and improve the quality of life of patients (Tanaka et al., 2008; Al-Moraissi et al., 2020). Although many clinical studies have investigated the effect of various treatments, no clinically approved therapeutics are currently available to restore the TMJ structure, given the limited understanding of its pathogenesis and the limited blood supply of the cartilage (Huey et al., 2012; Wang et al., 2015). Since obtaining clinical samples from patients with TMJOA is difficult and clinical symptoms often occur late in the disease process, animal models of TMJOA play a key role in understanding the pathogenesis of diseases and evaluating new therapeutic approaches (Vapniarsky et al., 2018; Liu et al., 2021). As various animal models of TMJOA have been developed over the past four decades, a major challenge lies in selecting the “best” model when designing a study. Animal models for TMD research and mouse genetic models for TMJ preclinical research have been reviewed elsewhere (Suzuki and Iwata, 2016; Ghassemi Nejad et al., 2017; Almarza et al., 2018; Bhatti et al., 2021; Xiang et al., 2021). This review serves to systematically summarize the usefulness, histopathological changes, and scope of application of each model and current animals used in TMJOA research. We hope to provide an evidence-based reference for researchers to deepen their understanding and to select appropriate TMJOA animal models.

## Characteristics of Temporomandibular Joint/Temporomandibular Joint Osteoarthritis

### Anatomy and Physiology of the Temporomandibular Joint

TMJ, a joint that connects the mandible to the skull and regulates mandibular movement, is composed of the mandibular condyle, articular disc, and the articular eminence and glenoid fossa. The cartilage layer on the mandibular condyle is from the superficial layer downward and composed of several layers: the fibrous, proliferative, hypertrophic and calcified cartilage layers (Thilander et al., 1976). Instead of being covered by the hyaline cartilage, the articular surface of the mandibular condyle is covered with a layer of mature fibrous tissue, consisting of a mass of collagen fibers (Toller, 1974). The hyaline cartilage is mainly composed of type II collagen, whereas the fibrocartilage is mainly composed of type I collagen (Vos et al., 2014). The orientation of the fibers on the condylar surface is a wavy interlacing of collagen fibers, which makes most fibers tangent to the surface (Toller and Wilcox, 1978). This property allows the TMJ to better withstand shear forces, whereas the hyaline cartilage is more resistant to compressive loading. Unlike the articular cartilage of the knee, the condylar cartilage has a different embryonic origin, which is derived from cranial neural crest cells (Shen and Darendeliler, 2005). Moreover, the most intriguing biological aspect of the condylar cartilage that differs from other cartilages lies in its ability to remodel in response to the changes in condylar repositioning, articular functioning, and mechanical loading (Nakano et al., 2003). Possibly, these essential structural differences in the TMJ significantly modifies the clinical expression of its pathological changes.

### Radiographical Features of Temporomandibular Joint Osteoarthritis

Several imaging techniques are available for TMJ visualization, including panoramic radiography, plain radiography, computed tomography (CT), magnetic resonance imaging (MRI), and high-resolution ultrasonography. Currently, CT and MRI are the most used imaging techniques (Talmaceanu et al., 2018). The radiographic manifestations of TMJOA include flattening of the anterior surface of the condyle, erosions, and irregularities of the joint surfaces, flattening of the articular surface of the temporal eminence, generalized sclerosis, subchondral cysts, osteophytes, and idiopathic condyle resorption (Zhao et al., 2011; Nah, 2012). Several studies suggest that erosive lesions may indicate acute or active changes, whereas sclerosis and flattening osteophytes may indicate a later and relatively stable stage (Ahmad et al., 2009).

### Histopathologic Features of Temporomandibular Joint Osteoarthritis

**FIGURE 1:**
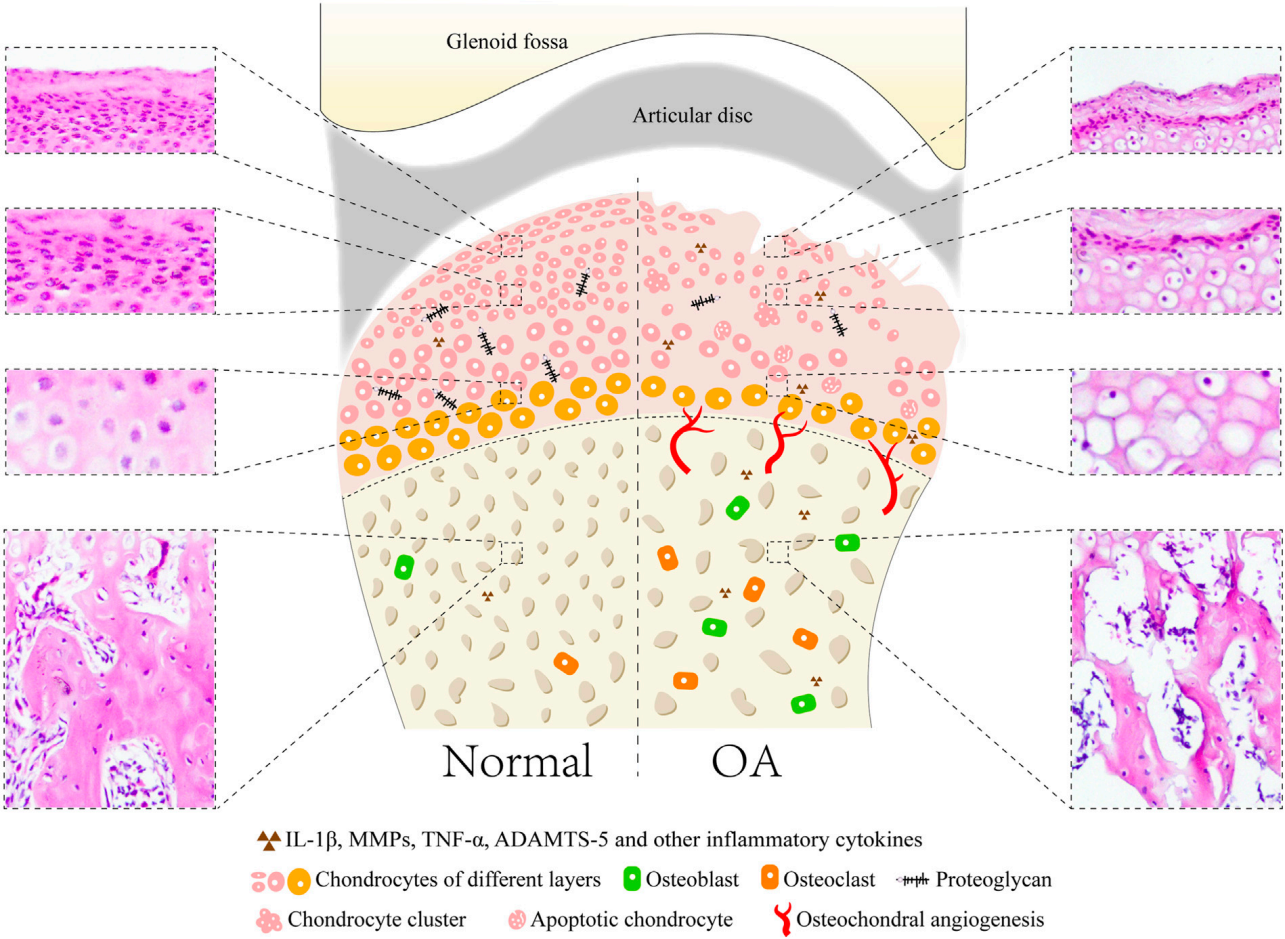
Common microstructural and histopathological alterations in cartilage and subchondral bone of TMJOA animal models with showing the normal joint and pathological joint. In normal joint, the surface of condylar cartilage is intact and smooth with four layers, including fibrous, proliferative, hypertrophic, and calcified cartilage layers. Lesions of cartilage include loss of cartilage surface integrity and proteoglycan, reduced and irregular arrangement of chondrocytes, presence of chondrocyte clusters and cell-free areas, decreased thickness of cartilage, apoptosis of chondrocytes and osteophyte formation. Subchondral bone appears as osteochondral angiogenesis, increased trabecular separation, large marrow cavities, decreased bone volume fraction, and activation of osteoblasts and osteoclasts.

The main manifestations of TMJOA are articular cartilage damage and degeneration, as well as repair of periarticular tissues and hyperplastic changes of the synovial membrane. Cartilage damage is characterized by irregular thinning and fibrillation of the fibrous layer, and reduced proteoglycan content of the cartilage matrix. Chondrocytes are frequently arranged in small groups or clusters, and many degenerated and necrotic chondrocytes are observed (de Bont et al., 1985a; de Bont et al., 1985b). In the superficial layers, the density of the collagen fibrils is diminished. The collagen fibrils show a loose and disordered arrangement. The calcified cartilage layer shows an irregular border adjacent to the fibrous layer and subchondral bone. Exposure of subchondral bone, hyperplasia, sclerosis, osteophyte formation and osteoblast activity can be found in subchondral bone (Toller, 1977) (Figure 1).

The synovial membrane of the TMJ may initially undergo synovial intima hyperplasia and cell hypertrophy, and subsequently result in deposition of fibrous material in the intima matrix. Subintimal fibroblast activity increases, and subintima elastic fibers are present (Dijkgraaf et al., 1997). Neovascularization of the fossa cartilage and articular disc frequently occur. The joint capsule is usually thickened in the TMJ. Adhesions to the lateral TMJ structures, including the synovial membrane, articular disc, and articular eminence, are often found in the latter stages of TMJOA (Dijkgraaf et al., 1995).

In summary, TMJOA is a chronic disease characterized by degenerative changes in the cartilage, accompanied by repair of surrounding tissues. Notably, TMJOA is different from OA in other synovial joints. Numerous elastic fibers, giant collagen fibrils, prominent nuclear fibrous lamina, and mineral-containing matrix vesicles are found in the degenerated condylar cartilage, which are not found in knee joint OA. Moreover, the inflammatory infiltrate is less often present in the osteoarthritic synovial membrane of the TMJ than in other synovial joints (Roy and Meachim, 1968; Meachim and Sheffield, 1969; de Bont et al., 1985b). The etiology and treatment of TMJOA are different because of differences in the structure and origin of cells that give rise to TMJ structures. Therefore, special animal models are needed to study TMJOA.

## Classification of Animal Models in Temporomandibular Joint Osteoarthritis

**FIGURE 2:**
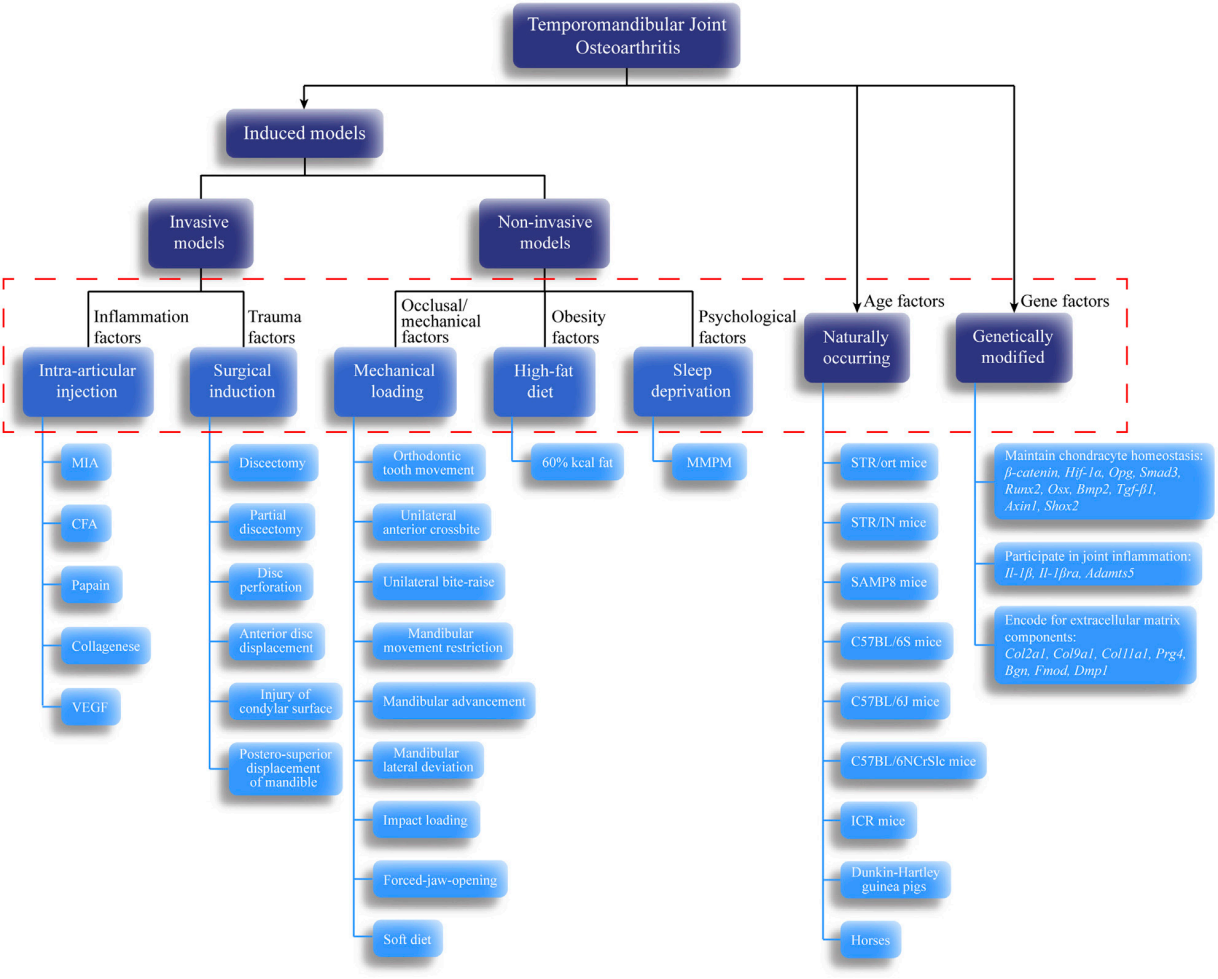
Classification of temporomandibular joint osteoarthritis (TMJOA) models. These models and their subdivisions share a relationship with TMJOA phenotypes. Black arrows indicate the classification based on whether the animals are treated with or without intervention. Dashed red box represents pathogenic factors simulated by each model. Blue lines indicate the specific animal species or model-building methods for each animal model.

Although some similarities in the disease processes between animals and humans are known, no one animal model is sufficient for studying all features of TMJOA. The translatability of animal models mainly depends on how well they correspond to human conditions. Therefore, we systematically summarized the existing animal models of TMJOA (Figure 2). Animal models used to study TMJOA are broadly divided into induced, naturally occurring, and genetically modified models, depending on whether the animals are treated with or without intervention. The induced models can be divided into invasive models (intra-articular injection and surgical induction) or non-invasive models (mechanical loading, high-fat diet, and sleep deprivation). Different modeling approaches mimic different etiologies of TMJOA. Selection of an appropriate animal model in studying TMJOA may be challenging. Therefore, we summarized the advantages, disadvantages, and indications of TMJOA animal models (Table 1).

**TABLE 1:** Common TMJOA models and their basic characteristics.

Model	Pros	Cons	Indications
Intra-articular injection models	Easy to operate	Pathogenic mechanism is different from human TMJOA	Mainly used for study of pain and inflammatory response
	Small trauma		
	Dose-dependent effects		
Surgical induction models	Induce TMJOA quickly	Risks of inflection	Mimic post-traumatic TMJOA
	Severe lesions	May affect other part of the joint	
Mechanical loading models	No trauma	Mild lesions	Mimic TMJOA caused by occlusal factors
	Present little risk for animals	Complex process of model-building	
		Need specialized equipment	
High-fat diet models	Easy to operate	Mild lesions	Mimic TMJOA affected by obesity factors
	High repeatability		
Sleep deprivation models	High repeatability	Sleep of rodent animals are naturally different from that of human	Mimic TMJOA under psychological stress
Naturally occurring models	No external interventions and induction required	Slow procession of disease	Mimic primary TMJOA
		Extremely long research period	
		High cost	
Genetically modified models	Develop disease naturally	Only act on specific genes	Study the function of a specific gene in TMJOA pathogenesis
		Long research period	
		High cost	

### Induced Models

#### Invasive Models

Invasive TMJOA models mainly work by producing joint destabilization, altered articular surface contact forces, and intra-articular inflammation in TMJs of animals. The procedures include injection and surgical approaches, which are related to high technique sensitivity. Therefore, improvement in the technical stability of researchers by long-term practice is the key to creating invasive models.

##### Intra-Articular Injection Models

Intra-articular injection is a well-characterized preclinical model of OA in the knee joint and TMJ. It causes disease by inducing intra-articular inflammation, cytotoxicity, or direct matrix damage in articular cartilage. Chondrocytes are the only cell type responsible for producing extracellular matrix and maintaining the homeostasis of cartilage (Dijkgraaf et al., 1995). Death of the chondrocytes, which results from necrosis or apoptosis, is a major feature of cartilage degeneration in OA (Aigner and Kim, 2002; Liu et al., 2021). Chemical drug injection can cause rapid death of many chondrocytes and destroy the homeostasis of chondrocytes, thereby creating joint damage and pain.

The commonly used drugs include monosodium iodoacetate (MIA) (Wang X. D. et al., 2012; Cledes et al., 2006; Guler et al., 2011), complete Freund’s adjuvant (CFA) (Rotpenpian et al., 2021; Xu et al., 2016; Xu et al., 2017), collagenase (Li W. et al., 2021; Li W. et al., 2014; Wu et al., 2015), papain (Molinet et al., 2020) and vascular endothelial growth factor (VEGF) (Shen et al., 2015) (Table 2). The first four drugs are common drugs in animal models of knee OA, which can cause different types of inflammation. The most frequently used drug among these drugs is MIA. However, a transcriptome study reported that ≤4% of total gene overlap occur between MIA-induced model and human OA (Barve et al., 2007). Despite this challenge, intra-articular injection still has the advantages of ease of induction and reproducibility. Additionally, the rate of disease progression and severity of joint lesions can be adjusted by changing drug concentration (Wang X. D. et al., 2012), which can provide acute disease model for researchers to design short-term studies.

**TABLE 2:** Intra-articular injection models of TMJOA animal models.

Drugs	Species	Changes of Condylar Cartilage	Changes in other parts of TMJ	Molecular Mechanisms
MIA	Rat (Wang et al., 2012c) Rabbit (Cledes et al., 2006; Guler et al., 2011)	Cartilage matrix degradation Fibrillation Chondrocyte apoptosis	Subchondral bone degradation Synovial hyperplasia Disc perforation Glenoid fossa degradation	↑ *Mmp-3, Mmp-13, Adamts-5*, *Tnf-α*, *Fas*, *Fasl*, *Bax*, *Caspase-8*, *Pcna*, *α-SMA* in whole condyle; MMP-3, CASPASE-3, α-SMA in hypertrophic layer ↓ *Aggrecan*, *Col1a1*, *Col2a1* and *Timp2* in whole condyle
CFA	Mouse (Rotpenpian et al., 2021) Rat (Xu et al., 2016; Xu et al., 2017)	Cartilage defection Cartilage matrix degradation	Subchondral bone degradation Bone remodeling Synovial hyperplasia	↑ RANKL, OCN, MMP-13, COL X, ADAMTS-5 in whole condyle; IHH, PTCH1 in hypertrophic layer; SMO, GLI1 in hypertrophic and mineralized layer ↓ OPG in whole condyle
Collagenase	Mouse (Li et al., 2021a) Rat (Li et al., 2014a) Rabbit (Wu et al., 2015)	Cartilage matrix degradation Endochondral ossification Increased chondrocyte synthesis	Subchondral bone degradation Bone remodeling Chondroid metaplasia Articular capsule hyperplasia	↑ *Cox-2*, *P65*, *Mmp-1, Mmp-13,* SOX-9, ADAMTS-5, MMP-9, COL II in whole condyle; CD44 in subchondral bone ↓ TIMP-3, *Col2a1* in whole condyle
Papain	Rabbit (Molinet et al., 2020)	Cartilage matrix degradation	Articular disc degradation Articular capsule degradation Decreased lower joint space	—
VEGF	Mouse (Shen et al., 2015)	Cartilage matrix degradation Fibrillation Chondrocyte apoptosis	Subchondral bone degradation Subchondral bone sclerosis	↑ MMP-9 and MMP-13 in hypertrophic layer; VEGFR2 in all cartilage layers; RANKL in subchondral bone

Mice, rats, and rabbits are widely used in intra-articular injection models. The most common animal models are rats (Wang X. D. et al., 2012; Li W. et al., 2014; Xu et al., 2016; Xu et al., 2017), because rats are easily managed and require low maintenance costs. Using radio-opaque dye, Hutchins et al. have demonstrated that the superior joint space of rats can hold 50–70 µl of injectable fluid (Hutchins et al., 2000). Therefore, the current dose of drug injection is approximately 50 µl. Kameoka et al. (2010) have tested three puncture techniques which are commonly used in humans for TMJ cavity in rats, and have found that the puncture success rate for anterosuperior puncture technique (ASPT) was significantly higher than others. Currently, different puncture techniques are in use (Cledes et al., 2006; Wang X. D. et al., 2012; George et al., 2013). Further studies are still needed to determine a standard procedure.

Intra-articular injection models are mainly used to investigate the molecular mechanisms of osteoarthritic pain and screening of preclinical therapies (Kim et al., 2019; Sannajust et al., 2019; Yi et al., 2020; Rotpenpian et al., 2021). Xu et al. (2017) suggested that IHH signaling facilitates TMJOA in CFA-induced rats by driving formation of hypertrophic chondrocytes and expression of catabolic enzymes, such as type X collagen, MMP-13, and ADAMTS-5, which may lead to degenerative changes in the articular cartilage. As an angiogenic factor, NETRIN-1 has been found to be a possible regulator during bone degeneration and pain in the process of TMJOA in MIA-induced mice (Xiao et al., 2021). Moreover, intra-articular injection may be appropriate for screening symptom-modifying OA drugs but not for disease-modifying OA drugs, because its pathophysiology is distinct from that of naturally occurring OA (Thote et al., 2013). Even so, intra-articular injection models still have the virtue of detecting joint pain-related mechanisms due to the rapid injuries occurring in the cartilage.

##### Surgical Induction Models

Surgery is the most widely used approach for building OA models. It can cause structural damage and abnormal articular forces to induce OA-like lesions directly by using surgical and mechanical devices. The common surgical methods include discectomy (Hinton, 1992; Lan et al., 2017; Liu X. et al., 2020; Saito et al., 2021), partial discectomy (Man et al., 2009; Xu et al., 2009; Lei et al., 2022), disc perforation (Embree et al., 2015; Luo et al., 2020; Ruscitto et al., 2020), anterior disc displacement (Togni et al., 2018; Xu et al., 2018; Nguyen et al., 2020), injury of the condylar surface (Ishimaru and Goss, 1992; Wang F. et al., 2017), and postero-superior displacement of the mandible (Imai et al., 2001; Liu et al., 2006) (Table 3). These six methods well mimic advanced symptoms of joint injury in clinical patients, but they are not starting factors in general TMJOA. Hence, surgical induction models are not appropriate for the study of the mechanisms of TMJOA, except in the conditions of direct trauma to the mandible.

**TABLE 3:** Surgical induction models of TMJOA animal models.

Surgical Induction Models	Species	Changes of Condylar Cartilage	Changes in other parts of TMJ	Molecular Mechanisms
Discectomy	Mouse (Liu et al., 2020b; Lan et al., 2017) Rat (Hinton, 1992) Rabbit (Saito et al., 2021)	Cartilage defection Cartilage matrix degradation	Subchondral bone degradation Diffuse osteochondral junction	↑ NOTCH1, HES5, TLR4, IL-1 β, TNF-α, ADAMTS-5, MMP-13 in cartilage; JAGGED-1 in chondrocyte clusters; NFκB P65, MyD88 in fibrous layer ↓ HES1 in whole condyle
Partial discectomy	Mouse (Xu et al., 2009; Lei et al., 2022) Rabbit (Man et al., 2009)	Cartilage defection Cartilage matrix degradation Fibrillation	Subchondral bone degradation Diffuse osteochondral junction Large marrow cavities	↑ DDR-2, MMP-13 in fibrous layer; IFN-γ, pSTAT4 in condylar cartilage ↓ *Aggrecan, Col2a1* in whole condyle
Disc perforation	Rat (Luo et al., 2020) Rabbit (Embree et al., 2015) Pig (Ruscitto et al., 2020)	Cartilage defection Cartilage matrix degradation Fibrillation Increased thickness of cartilage	Articular disc hyperplasia Articular disc calcification Diffuse osteochondral junction	↑ RUNX2, BSP in hypertrophic layer; CD31, α-SMA in cartilage ↓ COL I, COL II in hypertrophic layer
Anterior disc displacement	Rat (Togni et al., 2018; Nguyen et al., 2020) Rabbit (Xu et al., 2018)	Cartilage matrix degradation Cartilage hyperplasia Cartilage hypoplasia Osteophytes	Subchondral bone degradation Glenoid fossa degradation Articular disc deformity	↑ ADAMTS-5 in hypertrophic layer; CHOP, CASPASE-3, GRP78, *Caspase-12* in whole condyle
Injury of condylar surface	Sheep (Ishimaru and Goss, 1992; Wang et al., 2017a)	Cartilage matrix degradation Endochondral ossification Osteophytes	Subchondral bone degradation Articular disc perforation Glenoid fossa hyperplasia Synovial hyperplasia	—
Postero-superior displacement of mandible	Rabbit (Imai et al., 2001; Liu et al., 2006)	Cartilage defection Cartilage matrix degradation Osteophytes	Subchondral bone degradation Articular disc deformity Articular eminence hyperplasia Fibrous adhesion	—

Each surgical approach has its unique effect on joint mechanics and causes different changes in the biomechanical environment in the TMJ cavity. Therefore, the OA-like lesions they formed have different disease progression rates. When selecting an appropriate surgical approach, the anatomy and biomechanics of the selected animal TMJ, expected progression of the disease, and severity of late-stage lesions of animal models should be understood. This understanding allows researchers gain control of the entire course of disease development.

Mice, rats, rabbits, pigs, and sheep are widely used *in vivo* preclinical studies as surgical induction models. Rabbits are the most used models (Imai et al., 2001; Man et al., 2009; Embree et al., 2015; Xu et al., 2018; Saito et al., 2021), because they are quite large and strong enough to fight off infection. Therefore, they tolerate surgeries; their joint tissues are large enough to undergo surgery and wear mechanical devices. In addition, rabbits are easier to operate and cheaper than large animals, such as pigs and sheep.

Surgical induction models are mainly used for tissue engineering, stem cell transplantation, and local growth factor treatment studies (Ying et al., 2013; Embree et al., 2016; Tarafder et al., 2016; Wang KH. et al., 2017). Embree et al. (2016) discovered that resident fibrocartilage stem cells (FCSCs) localized within the fibrous layer possess potent chondrogenic and osteogenic potential. Additionally, they suggested that regulation of canonical Wnt signals can sustain FCSC pool and maintain tissue homoeostasis, which provide new concepts on the development of potential therapies for TMJ regeneration. Before these regenerative strategies can be applied to humans, future studies using preclinical animal models are still required to include long-term cartilage and bone structure recovery, as well as biomechanical analyses, to verify that functional joint recovery is achieved. Therefore, surgical induction models are more suitable for osteochondral interface repair investigations, given the directly damaged TMJ structure.

One problem with many invasive models, however, is that they only operated on one side of the TMJ and use the other side as controls (Shinoda et al., 2005; Embree et al., 2015; Lemos et al., 2016). Unlike most other synovial joints, TMJ is bilaterally linked, and mastication, mouth opening, and other actions need to be completed together. Therefore, when using unilateral intervention methods, the influence on the other joint should be considered (Cohen et al., 2014). In recent years, various methods have been improved to create models while preserving as much tissue as possible in TMJ to prevent the effect of the surgical procedure on animals (Gu et al., 2006; Nguyen et al., 2020). In addition, different surgical approaches used in the same model will cause different pathological changes. Therefore, further studies on invasive models are needed in the future to achieve better model establishment.

#### Non-Invasive Models

Non-invasive models cause joint injury by applying external mechanical force, high fat diet, or mental stimulation without causing open trauma or articular capsule damage. In this way, the model-building process is completely sterile, and the effect of the invasive models on remaining joint tissues is eliminated. In addition, no surgical procedures are required on animals because such models mainly use mechanical devices to assist modeling.

##### Mechanical Loading Models

Appropriate stress stimulation can promote chondrocyte proliferation and extracellular matrix synthesis. However, when damage caused by mechanical loading exceeds the joint’s ability to repair itself, the affected joint would suffer damage, and even develops into OA (Tanaka et al., 2008; Liu et al., 2021). The functional movement and biomechanical loading of TMJ are closely related to occlusion. Abnormal dental occlusion is one of the potential causes of TMJOA, which includes severe malocclusion and skeletal jaw asymmetry. Thus, the TMJOA models can be built by disordered occlusion, which is the TMJ-specific model-building approach, including orthodontic tooth movement (Wang Q. Y. et al., 2012; Zhang et al., 2013), unilateral anterior crossbite (Wang Y. L. et al., 2014; Zhang et al., 2016; Liu J. et al., 2020), unilateral bite-raise (Long et al., 2019; Ou et al., 2021), mandibular movement restriction (Teramoto et al., 2003; Li et al., 2013), mandibular advancement (Yang et al., 2020; Li Y. et al., 2021), and mandibular lateral deviation (Zhao et al., 2010; Zou et al., 2022). Injury to the TMJ can be caused by indirect force to the mandible, which may lead to local pain, dislocation, or fracture, even TMJOA. Thus, impact loading can be used to establish the TMJOA model (Wang et al., 2008). Mastication provides a crucial mechanical stimulus for jawbone remodeling. Sufficient loading is important in maintaining the appropriate proliferation of chondrocytes and matrix production in the condyle. Thus, muscle overuse or underuse, such as forced-jaw-opening (Fujisawa et al., 2003; Nicoll et al., 2010; Khurel-Ochir et al., 2021) and soft diet (Ikeda et al., 2014; Robinson et al., 2019), can be used to establish TMJOA models by affecting the metabolism of the condyle. The above models simulated TMJOA caused by occlusal factors in clinical patients (Table 4).

**TABLE 4:** Mechanical loading models of TMJOA animal models.

Mechanical loading models	Species	Changes of Condylar Cartilage	Changes in other parts of TMJ	Molecular Mechanisms
Orthodontic tooth movement	Rat (Wang et al., 2012b; Zhang et al., 2013)	Cartilage matrix degradation Chondrocyte autophagy in hypertrophic layer Endochondral ossification Osteophytes	Subchondral bone degradation Bone remodeling Osteochondral angiogenesis	↑ CTXs in serum; RUNX-2, VEGF, CTGF, MMP-9, CHM-1, M-CSF, RANKL/OPG in hypertrophic layer; BECLIN-1, LC3-II in whole condyle ↓ OPG, MAP4K3 in hypertrophic layer; p-MTOR, p-P70S6 K in whole condyle
Unilateral anterior crossbite	Mouse (Liu et al., 2020a) Rat (Wang et al., 2014b; Zhang et al., 2016)	Cartilage matrix degradation Chondrocyte apoptosis in hypertrophic layer Mineral deposition	Subchondral bone degradation Neomineralization	↑ GRP78, CHOP, CASPASE-12, cleaved-CASPASE-3, *Tnap, Mmp-13* in cartilage ↓ PCNA, COL II, COL X in hypertrophic layer; *CD73, Npp1* in cartilage ATF6, *Derlin-3*, MMP-9, TIMP-1, MGP first increase and then decrease
Unilateral bite-raise	Mouse (Ou et al., 2021) Rat (Long et al., 2019)	Fibrillation Cartilage matrix degradation Inflammation	Subchondral bone degradation	↑ IL-6, TH in hypertrophic layer; IHH, SMO, MMP-13, CASPASE-3 in proliferative and hypertrophic layers; GLI-1 in cartilage ↓ TH in fibrous and proliferative layers
Mandibular movement restriction	Rat (Teramoto et al., 2003, Li et al., 2013)	Cartilage matrix degradation Chondrocyte apoptosis in hypertrophic layer	Subchondral bone degradation Local bone sclerosis Bone remodeling	↑ BRDU in proliferative layer; Op, PDI, CRT, CHOP, CASPASE-3, BIP, p-EIF2α in cartilage ↓ COL II, COL X, p-PIN1, TCTP, *Runx2* in whole condyle
Mandibular advancement	Rat (Yang et al., 2020; Li et al., 2021b)	Cartilage matrix degradation Chondrocyte apoptosis	Subchondral bone degradation Bone remodeling	↑ MMP-13, CXCR4, SDF-1 in hypertrophic layer; RUNX2 in cartilage; OSX, p-S6 in subchondral bone ↓ COL II in cartilage
Mandibular lateral deviation	Rat (Zou et al., 2022) Rabbit (Zhao et al., 2010)	Cartilage defection Cartilage matrix degradation	Subchondral bone degradation Myelofibrosis Bone remodeling	↑ *Mmp8, Ifit1, Ifit3, Itgb1, Itgb3*, ITGB2, VEGF in cartilage; nNOS in synovial membrane ↓ *Sox9, Itgb4* in cartilage; SOD in synovial membrane
Impact loading	Goat (Wang et al., 2008)	Cartilage defection Cartilage matrix degradation Osteophytes	Exposure of subchondral bone Synovial hyperplasia Fibrous adhesion Articular disc defection	↑ MMP-3 in cartilage; TIMP-1 in hypertrophic layer and synovial membrane MMP-13 first increase and then decrease
Forced-jaw-opening	Mouse (Khurel-Ochir et al., 2021) Rat (Nicoll et al., 2010) Rabbit (Fujisawa et al., 2003)	Fibrillation Cartilage matrix degradation Chondrocyte apoptosis Increased blood vessel and multinucleated osteoclasts in hypertrophic layer	Exposure of subchondral bone Bone remodeling Increased mechanical sensitivity Articular disc degradation	↑ MMP-1, MMP-3, MMP-9, MMP-13, IL-1β, *Caspase-3*, VEGF in proliferative and hypertrophic layers ↓ ACAN in cartilage
Soft diet	Mouse (Robinson et al., 2019) Rat (Zhang et al., 2021a)	Cartilage matrix degradation Chondrocyte apoptosis	Subchondral bone degradation Decreased bite force	↑ MMP-3, MMP-13 in whole condyle; *Prg4* in cartilage ↓ COL 2, *Pthrp, Ihh, Col Ⅹ* in cartilage

The most common animals used for mechanical loading models are rats (Teramoto et al., 2003; Li et al., 2013; Long et al., 2019; Yang et al., 2020; Li Y. et al., 2021; Zou et al., 2022), because rats are common rodents and can tolerate the installation of mechanical devices. However, due to the significant differences in occlusal and TMJ structure between rats and human, OA-like lesions induced by the mechanical loading method are not completely equivalent to human TMJOA lesions (Wang et al., 2015). Given the high similarity between mechanical loading models and human TMJOA caused by occlusal factor, larger animals with a more similar structure to human TMJ, such as pigs and sheep, should be used in future studies on pathogenesis.

Currently, mechanical loading models are mainly applied to study the mechanism of pathological changes in TMJOA (Jiao et al., 2009; Jiao et al., 2011; Ma et al., 2020; Ou et al., 2021), probably because this kind of model directly mimics the disease process in TMJOA patients caused by disordered occlusion. The pathogenic mechanism of TMJOA in mechanical loading models has been widely discussed. Zhang et al. (2022) revealed that elevated expression of SEMA4D in early-stage TMJOA might decrease the bone formation activity of osteoblasts in the subchondral bone by binding to PLEXIN-B1 expressed by osteoblasts. HIF-1, which may repress OPG expression, was activated in mature chondrocytes in mechanical loading models, resulting in osteoclastogenesis and development of TMJOA (Shirakura et al., 2010). He et al. (2018) discovered several new genes that had never been reported to be associated with TMJOA by RNA sequencing. These genes may be used as potential therapeutic genes related to TMJOA. In the future, attention should be paid to the development of therapeutic strategies that take full advantage of this model.

##### High-Fat Diet Models

Obesity is found to be associated with OA. Overweight and obesity do not only significantly increase the risk of incident hip and knee OA, but also aggravate its radiographic changes (Johnson and Hunter, 2014). Studies in experimental animals have shown that obesity increases the incidence and severity of OA (Issa and Griffin, 2012). According to the cross-sectional studies, obesity is also associated with TMJ disease (Jordani et al., 2017; Karaman and Sadry, 2021). In addition, several chewing characteristics, such as chewing speed and duration, are associated with obesity in young adolescents, and they might affect the development of the TMJ. Therefore, further investigation is needed to reveal the relationship among jaw mastication, obesity, and TMJOA.

Griffin et al. (2010) first observed loss of proteoglycans in the TMJ of C57BL/6J mice with a high-fat diet (45% kcal fat) for 45 weeks. Du et al. (2020) studied the effect of high-fat diet (60% kcal fat) on TMJ of C57BL/6 mice. Less cartilage matrix, thinner condylar cartilage, and vertical clefts were observed in overweight mice after 12 weeks of high-fat feeding. Additionally, they found that the expression of IL-1β, MMP-3 and leptin were upregulated in condylar cartilage of high-fat-fed mice. Patients with knee OA have increased level of serum leptin and the abnormal leptin level in synovial fluid. Leptins are one of the increased proinflammatory factors in individuals with obesity (Conde et al., 2013). This research team also confirmed that statins had anti-inflammatory effects in TMJOA-like changes and a protective effect on the damaged TMJ cartilage.

Not only does diet induce obesity-increased OA joint pathology in mice but also induces anxiety and hyperalgesia, and reduces muscle function and locomotor activity (Griffin et al., 2010). Currently, studies only focus on the establishment of high-fat TMJOA model, and more investigations will be required in the future to reveal the mechanisms involved in obesity-induced TMJOA.

##### Sleep Deprivation Models

Osteoarthritis is one of the characteristics of premature aging in organisms. It is affected by circadian disturbances (Berenbaum and Meng, 2016). Many studies have shown that psychological factors, such as sleep disorders, mental stress, and depression, may be related to TMJ dysfunction (LeResche et al., 2007; Slade et al., 2007). Therefore, establishment of sleep deprivation models could be helpful for related studies. This model mainly applies the modified multiple platform method (MMPM) proposed by Suchecki and Tufik (2000). The principle of this technique is to take advantage of the rat’s fear of water and inability to sleep in water. A certain number of platforms with small diameters (≤6.5 cm in diameter) are placed in a tank filled with water. The rats can stand on the platform and jump between the platforms. When the rats are about to sleep, their muscles relax and their faces would touch the water, which could awake them to achieve the goal of sleep deprivation in rats.

Chen et al. (2013) first showed that the surface of fibrous layer was cracked and exfoliated in sleep-deprived rats, compared with control rats, suggesting that sleep deprivation may lead to histopathological changes in the TMJ of rats. Chen et al. (2020) then successfully built a TMJOA model by using a similar model and demonstrated that sleep deprivation could induce OA-like lesions in TMJ of rats, and the OA-like lesions may be reversible in the early stage. Additionally, they found that rhythmic gene expression dysregulation in sleep deprivation models, which further leads to MAPK/ERK signaling pathway activation and then aggravates TMJOA. Chen Y. et al. (2019) indicated that hypoxia played an important role in TMJOA and accelerated angiogenesis of condylar cartilage through the HIF-1-VEGF-Notch signaling pathway. These studies may provide new insights into the clock gene mechanism of endochondral homeostasis and the complex pathophysiological mechanism of TMJOA.

Currently, this model only applies to rats, and the therapeutic effect of low intensity pulsed ultrasound (LIPUS) on this model has been studied. Liang et al. have found that LIPUS had a good treatment effect on early TMJ injury by regulating the MMP-3/TIMP-1 and RANKL/OPG expression ratios in cartilage tissues, and have demonstrated that LIPUS treatment at an intensity of 45 mW/cm^2^ for at least 2 weeks is the optimal regimen for TMJOA in rats (Liang et al., 2019; Liang et al., 2020). Given the difficulty of using humans as participants to advance this study, the establishment of an experimental animal model of TMJOA is necessary to further study the pathogenesis of TMJOA under psychological stress, especially in studies for testing clinical treatment and exploring better medications.

### Naturally Occurring Models

Some animals develop OA-like lesions with slow progression, which is very similar to the disease progression of primary OA in humans. Therefore, such models are often referred to as naturally occurring models. Studies have shown that STR/Ort (Kumagai et al., 2015; Yamashita-Futani et al., 2021), STR/IN (Dreessen and Halata, 1990), SAMP8 (Ishizuka et al., 2014), C57BL/6S (Fukuoka et al., 1993), C57BL/6J (Cui et al., 2020), C57BL/6NCrSlc (Ukita et al., 2020), ICR (Silbermann and Livne, 1979; Livne and Silbermann, 1986) mice, Dunkin-Hartley guinea pigs (Wu et al., 2016), and horses (Smyth et al., 2019) all manifest OA-like lesions with increasing age, among which multiple subtypes of SAM mice can develop OA-like lesions in TMJ (Chen et al., 1989) (Table 5). Yamashita-Futani et al. (2021) found in STR/Ort mice that the production of elastin-digested peptides was related to the upregulation of pro-inflammatory mediators, such as IL-6 and MMP-12. IL-6 induced the expression of ADAMTS-4 and ADAMTS-5 in chondrocytes, following cartilage degradation. OA lesions also appeared in articular cartilage of C57B/6S mice and were correlated to increased levels of collagen-like peptidase and prolyl endopeptidase in the serum, which indicated collagen degradation (Fukuoka et al., 1993). Ishizuka et al. revealed that a downregulation of IHH signaling accompanies the early onset TMJ degeneration changes in senescence-accelerated mice (Ishizuka et al., 2014). Naturally occurring models are ideal for studying cartilage degradation and bone remodeling in TMJOA and can provide evidence for the study of pathogenesis of TMJOA at different ages. The induced model-building methods can also be used to cause diseases in such animals, which can naturally result in TMJOA to study the effect of external stimulus during the disease course of primary TMJOA.

**TABLE 5:** Naturally occurring models of TMJOA animal models.

Strain of animals	Age of Onset	Changes of Condylar Cartilage	Changes in other parts of TMJ	Molecular Mechanisms
STR/Ort mice (Kumagai et al., 2015; Yamashita-Futani et al., 2021)	40-week-old	Cartilage defection Cartilage matrix degradation	Subchondral bone degradation Subchondral bone resorption Intramembranous ossification	↑ MMP-12 in cartilage; IL-6, ADAMTS-4, ADAMTS-5 in subchondral bone
STR/IN mice (Dreessen and Halata, 1990)	36-week-old	Cartilage defection Cartilage matrix degradation Increased lysosomes in chondrocytes	Glenoid fossa degradation Synovial metaplasia Decreased lower joint cavity No inflammation in synovial membrane	—
SAMP8 mice (Ishizuka et al., 2014)	16-week-old	Cartilage matrix degradation Increased thickness of cartilage	—	↓ *Col1a1, Col2a1* in cartilage; *Col10a1, Ihh, Gli1, Gli2, Ptch1, Hip* in whole condyle
C57BL/6S mice (Fukuoka et al., 1993)	12-week-old	Cartilage defection Cartilage matrix degradation Osteophytes	Subchondral bone degradation Synovial hyperplasia	↑ CL-peptidase, PEP in serum
C57BL/6J mice (Cui et al., 2020)	45-week-old	Cartilage defection Cartilage matrix degradation	Subchondral bone degradation Bone remodeling	↑ MMP-13, COL Ⅹ in cartilage; P16^ink4a^, pSMAD3, CTSK in subchondral bone ↓ COL I, RUNX2, OSX in subchondral bone
C57BL/6NCrSlc mice (Ukita et al., 2020)	80-week-old	Cartilage defection Cartilage matrix degradation	Subchondral bone degradation	↓ H3K9Me1, H3K9Me2, H3K9Me3 in hypertrophic layer
ICR mice (Silbermann and Livne, 1979; Livne and Silbermann, 1986)	28-week-old	Cartilage defection Cartilage matrix degradation	Subchondral bone degradation Focal ankylosis between condyle and articular disc	—
Dunkin-Hartley guinea pigs (Wu et al., 2016)	12-week-old	Cartilage matrix degradation	Large marrow cavities Bone remodeling Glenoid fossa remodeling	↑ CAD-11, MMP-3 in proliferative and hypertrophic layer of cartilage ↓ COL II in proliferative and hypertrophic layer of cartilage
Horses (Smyth et al., 2019)	—	Cartilage matrix degradation	Articular disc degradation Articular disc metaplasia	—

The naturally occurring models have slow disease progression. Like the spontaneous OA-like lesions in human TMJ, naturally occurring models do not require invasive procedures to generate the arthritis, thus eliminating many potential side effects. They are thought to be closely related to the natural progression of TMJOA in humans, which are inapplicable for simulating the development of post-traumatic TMJOA. Since obtaining the articular cartilage samples of TMJOA in humans is difficult, naturally occurring models have gradually served as important models for the study of pathogenesis of OA. The underlying mechanisms that drive the onset and progression of spontaneous TMJOA in these animals are not well defined and may reflect specific subtypes of idiopathic human TMJOA. Currently, few studies have been conducted on this type of model. Due to the extremely long study period and high cost, most of these studies focus on the etiological mechanism.

### Genetically Modified Models

The use of genetically modified mice has greatly improved our understanding of the precise molecular pathophysiology and therapies of many human diseases (Little and Hunter, 2013). In the field of TMJOA, specific genetic modifications are made to the mice to reveal the role of different genes in TMJ development or disease processes. Unlike invasive animal models, genetically modified models can provide biological information for a population that is prone to developing TMJOA. Since genetic and environmental factors can be precisely controlled, this kind of model has the potential to reveal molecular pathways involved in the progressive degeneration of TMJ.

Preclinical studies of genetically modified mice have increased over the past two decades, making them the best candidate models for the study of the molecular pathway involved in TMJOA (Table 6). The genes involved in this review can be divided into three main categories. The first group mainly maintained chondrocyte homeostasis; they include transcription factors or signaling regulators (*β-catenin*, *Hif-1α*, *Opg*, *Smad3*, *Runx2*, *Osx*, *Bmp2*, *Tgf-β1*, *Axin1*, *Shox2*), enzymes [*1α*(*OH*)*ase*, *Dnmt3b*], and receptors (*Fgfr3*, *Bmpr1α*, *Ddr1*, *Ddr2*). Genes that participate in joint inflammation are the second group, including cytokine (*Il-1β*), receptor (*Il-1βra*) and enzyme (*Adamts5*). The third group includes genes encoding for extracellular matrix components (*Col2a1*, *Col9a1*, *Col11a1*, *Prg4*, *Bgn*, *Fmod*, *Dmp1*). The genes and gene products identified in genetically modified models as decreasing or increasing the severity of TMJOA-associated cartilage erosion can all be considered potential therapeutic targets. Appropriate inhibitors of these proteins and activators or recombinant versions may lead to the development of new therapies, which need to be further investigated.

**TABLE 6:** List of genes of genetically modified models.

Protein	Mice model	Changes of Condylar Cartilage	Changes in other parts of TMJ	Molecular Mechanisms
Genes encoding regulators of chondrocyte homeostasis				
Beta cadherin associated protein	*β-catenin(ex3)* ^*Col2ER*^ (Wang et al., 2014a)	Cartilage defection Cartilage defection	Subchondral bone sclerosis Decreased joint space	↑ COL X in hypertrophic layer; RUNX2, *Mmp-13, Adamts-4, Adamts-5* in cartilage ↓ COL II in hypertrophic layer
	*β-catenin(ex3)* ^*Agc1CreER*^ (Hui et al., 2018)	Cartilage matrix degradation Endochondral ossification Chondrocyte apoptosis	Subchondral bone sclerosis	↑ MMP-13 in fibrous, hypertrophic layer and articular disc; COL X in cartilage; ADAMTS-4, ADAMTS-5 in fibrous layer ↓ PCNA in cartilage; KI67 in proliferative layer
Hypoxia-inducible transcription factor 1α	*Hif-1α* ^*+/-*^ (Mino-Oka et al., 2017)	Cartilage matrix degradation Cartilage matrix degradation	—	↑ MMP-9, cleaved-CASPASE-3 in hypertrophic layer ↓ ACAN, VEGF, *Vegf* in condyle
	*Hif-1α* ^*fl/fl*^ *; ctsk cre+* (Tang et al., 2020)	Cartilage defection Cartilage defection	Decreased osteogenesis and angiogenesis in subchondral bone	↑ CASPASE-3 in cartilage; OCN in subchondral bone ↓ COL II, COL X in hypertrophic layer; VEGF, CD31, TRAF5, *Ctsk* in cartilage; MMP-9 in subchondral bone
Osteoprotegerin	*Opg* ^*-/-*^ (Chen et al., 2019a)	Cartilage matrix degradation Increased chondrocyte apoptosis Decreased chondrocyte proliferation	Subchondral bone degradation Diffuse osteochondral junction	↑ COL X in cartilage
Mothers against decapentaplegic homolog 3	*Smad3* ^*-/-*^ (Mori et al., 2015)	Fibrillation Cartilage matrix degradation Chondrocyte apoptosis	Subchondral bone degradation	↑ MMP-9, MMP-13, CASPASE-3, CASPASE-9 in cartilage ↓ p-SMAD3 in fibrous layer; COL II, ACAN, SPHK1, S1P_3_ in cartilage
Runt-related transcription factor-2	*Runx2* ^*fl/fl*^ *; Agc1-CreER* (Liao et al., 2019)	Cartilage defection Cartilage matrix degradation	—	↓ COL X, PCNA, IHH in hypertrophic layer; *Mmp-13, Col2a1, Acan* in cartilage
Osterix	*Osx* ^*fl/fl*^ *; Agc1-CreER* (Jing et al., 2014a)	Cartilage defection Increased cartilage matrix Decreased proliferation and apoptosis	Subchondral bone degradation Diffuse osteochondral junction Intramembranous ossification	↑ COL II, COL X, ACAN, SOX9 in hypertrophic layer ↓ VEGF, DMP1 in subchondral bone
Bone morphogenetic protein 2	*Bmp2* ^*fl/fl*^ *;Agc1-CreER* ^*T2*^ (O'Brien et al., 2021)	Cartilage matrix degradation	Decreased mineralization Decreased bone remodeling	↑ AMADTS-4, MMP-13 in cartilage
Transforming growth factor β1	*Tgf-β1 mutant* (Jiao et al., 2014)	Cartilage defection Cartilage matrix degradation	Subchondral bone degradation Local sclerosis in subchondral bone Subchondral bone resorption	↑ VEGF, MMP-9, MMP-13, CASPASE-3 in hypertrophic
Axis inhibition protein 1	*Axin1* ^*Agc1ER*^ (Zhou et al., 2019b)	Cartilage defection Cartilage matrix degradation Increased chondrocyte apoptosis Decreased chondrocyte proliferation	Subchondral bone sclerosis	↑ MMP-13, ADAMTS-5 in superficial layer; CATNB, *Col10a1, Fgfr1, Fgfr2, Fgfr3*, pERK1/2 in cartilage ↓ COL X in cartilage; Prg4, PCNA in superficial layer
Short stature homeobox 2	*Wnt1-Cre; pMes-stop Shox2* (Li et al., 2014b)	Cartilage dysplasia	Glenoid fossa dysplasia Chondrocyte apoptosis in glenoid fossa	↑ MMP-9, MMP-13 in cartilage ↓ COL I in glenoid fossa; COL II, IHH, GLI2 in condyle
	*Shox2* ^*SHOX-KI/KI*^ (Li et al., 2014c; Liang et al., 2016)	—	Chondrocyte apoptosis in articular disc	↑ MMP-9, MMP-13 in articular disc; COL I, MMP-9, MMP-13 in condyle ↓ COL I, ACAN in articular disc; IHH, COL II in condyle
1α-hydroxylase	*1α(OH)ase* ^*-/-*^ (Shen et al., 2013)	Cartilage defection Cartilage matrix degradation Chondrocyte apoptosis	Subchondral bone degradation Subchondral bone resorption	↑ 8-OHDG, γH2AX, β-GAL, p16^INK4A^, *Il-1α, Il-1β, Il-6, Mmp-3, Mmp-13, Adamts-5, Ctsk*, IL-1α, IL-1β, IL-6, MMP-3, MMP-13 in cartilage ↓ COL II in cartilage
DNA (cytosine 5)-methyltransferase 3 beta	*Dnmt3b* ^*fl/fl*^ *; Agc1-Cre* ^*ERT2*^ (Zhou et al., 2019a)	Cartilage defection Fibrillation Cartilage matrix degradation	—	↑ KI67, COL X, CATNB in cartilage ↓ COL II in cartilage
Fibroblast growth factor receptor 3	*Fgfr3* ^*P244R*^ (Yasuda et al., 2012)	Cartilage defection Cartilage matrix degradation	Subchondral bone resorption Articular disc fusion to the temporal bone	↓ *Ihh, Ptch1, H4C, Col2a1, Col10a1* in cartilage; *Col1a1, Mmp-13* in subchondral bone; *Ihh, Col1a1, Op* in secondary cartilage
	*Fgfr3* ^*fl/fl*^ *; Col2a1-CreER* ^*T2*^ (Zhou et al., 2016)	Cartilage defection Cartilage matrix degradation Chondrocyte apoptosis	Subchondral bone sclerosis No change in bone remodeling	↑ COL X, MMP-13, ADAMTS-5 in fibrous layer; IHH, RUNX2 in cartilage ↓ PRG4 in fibrous layer
Bone morphogenetic protein receptor-1A	*Bmpr1a* ^*fl/fl*^ *; Agc1-Cre* ^*ER*^ (Jing et al., 2014b)	Cartilage disappear and then appear Decreased chondrocyte proliferation	Subchondral bone sclerosis	↓ COL II, COL X, SOX9, in cartilage; OSX in subchondral bone
Discoidin domain receptor 1	*Ddr1* ^*-/-*^ (Schminke et al., 2014)	Cartilage defection Fibrillation Cartilage matrix degradation	Subchondral bone degradation	↑ COL I, COL IX, *Col10a1, Runx2* in cartilage ↓ COL II, NID-2, *Col3a1, Acan, Sox-9* in cartilage
Discoidin domain receptor 2	*Ddr2* ^*slie/slie*^ (Ge et al., 2018)	Cartilage defection Cartilage matrix degradation	Subchondral bone degradation Delayed mineralization in glenoid fossa and subchondral bone	—
Genes encoding inflammation mediators				
Interleukin-1β	*Col1-Il-1β* ^*XAT*^ (Lai et al., 2006; Huang et al., 2013)	Cartilage defection Fibrillation Cartilage matrix degradation	Presence of pain	↑ COL II, MMP-9, IL-6, COX-2, TGF-β in hypertrophic layer; NGF, TRKAR in cartilage
	*Il-1βra* ^*-/-*^ (Tabeian et al., 2019)	Cartilage matrix in fibrous layer first increase and then decrease	Subchondral bone degradation	—
A disintegrin and metalloproteinase with thrombospondin motifs 5	*Adamts5* ^*-/-*^ (Rogers et al., 2018; Rogers-DeCotes et al., 2021)	Cartilage matrix degradation Chondrocyte apoptosis in hypertrophic layer	Subchondral bone degradation Bone marrow infiltration	↑ ACAN in cartilage ↓ COL II, COL X in hypertrophic layer; *Sox9*, MMP-13 in cartilage; BGLAP in subchondral bone
Genes encoding components of the extracellular matrix				
Type II collagen	*Dmm/+* (Ricks et al., 2013; Long et al., 2016)	Cartilage defection Cartilage matrix degradation	Diffuse osteochondral junction	↑ TGF-β1, p-SMAD2, HTRA1 in chondrocytes; MMP-13, DDR2 in cartilage
	Del1 mice (Rintala et al., 1997)	Cartilage defection Cartilage matrix degradation	Subchondral cysts Fibrous adhesion Diffuse osteochondral junction	—
Type IX collagen	*Col9a1* ^*-/-*^ (Lam et al., 2007; Polur et al., 2010)	Cartilage defection Fibrillation Cartilage matrix degradation	—	↑ HTRA1, MMP-13 in fibrous layer; DDR2 in cartilage; MMP-derived type II collagen fragments in fibrous layer
Type XI collagen	*Col11a1* ^*+/-*^ (Lam et al., 2007; Polur et al., 2010; Long et al., 2016)	Cartilage defection Fibrillation Cartilage matrix degradation	—	↑ HTRA1 in fibrous layer; DDR2, MMP-13 in cartilage; TGF-β1, p-SMAD2, HTRA1 in chondrocytes; MMP-derived type II collagen fragments in superficial layer
Proteoglycan-4	*Prg4* ^*-/-*^ (Hill et al., 2014; Koyama et al., 2014)	Cartilage defection Cartilage matrix degradation Fibrillation	Subchondral bone resorption Articular disc hyperplasia Synovial hyperplasia Synovial infiltration	↑ COL II, COL X in hypertrophic layer; CTSK in subchondral bone; SOX-9 in cartilage; HAS-2 in cartilage, glenoid fossa, and synovial membrane
Biglycan Fibromodulin	*Bgn* ^*-/-*^ *; Fmod* ^*-/-*^ (Wadhwa et al., 2005; Embree et al., 2010)	Cartilage defection Chondrocyte apoptosis Osteophytes	Articular disc disruption Osteophytes in glenoid fossa	↑ COL I, COL II in fibrous layer ↓ ACAN in cartilage PCNA first decrease and then increase
Dentin matrix protein 1	S89G-DMP1 mice (Weng et al., 2017)	Cartilage defection Cartilage matrix degradation	Subchondral bone degradation Chondrocyte apoptosis in subchondral bone	↑ MMP-13, CASPASE-9 in cartilage ↓ COL I, COL II, ACAN, DCN, SOX9, PCNA, *Tgfb1, Alk1, Alk5, Smad1, Smad2, Smad3, Smad5, Smad9* in cartilage

Xu et al. found increased expression of DDR2 and increased level of proteoglycans in early TMJOA in both *Col9a1* ^*−/−*^ and *Col11a1* ^*−/−*^ mice (Lam et al., 2007). Over time, the chondrocytes synthesize and release matrix-degrading enzymes that degrade proteoglycans. One of the consequences of proteoglycan degradation is to enhance the exposure of chondrocytes to type II collagen fibrils, which may result in the activation of DDR2. The activation of DDR2 induces the expression of MMP-13, which then cleaves type II collagen. This eventually leads to the irreversible destruction of the articular cartilage. Therefore, chondrocyte clusters and increased proteoglycan production in the pericellular matrix have also been identified as early OA indicators.

Although the mouse models cannot simulate the biomechanical function of human joints, it is a major option for molecular studies. This is due to the advances in mouse genetics, and the easy availability of genetically modified mice, allowing the evaluation of time-dependent changes in TMJOA after specific genetic modifications. Moreover, genetically modified models have the virtue of eliminating other interferences to allow researchers directly observe how individual genes influence the process of TMJOA at the genetic level. Consequently, this kind of model facilitates the study and establishment of the molecular basis for TMJOA development. However, multiple genes are generally implicated in the pathogenesis of human TMJOA (Pitsillides and Beier, 2011), whereas genetically modified models mainly act on specific genes. Therefore, this model cannot comprehensively simulate the situation of TMJOA induced by multi-gene interaction. As with naturally occurring models, researchers need consider the lengthy experimental time and the cost of housing these animals.

## Current Animals Used in Temporomandibular Joint Osteoarthritis Research

Animal models are the primary means of testing potential therapeutic agents to determine their potential efficacy in TMJOA. However, existing animal models are inadequate to simulate complex clinical conditions. On the one hand, most animal models of TMJOA are single-factor models, which only simulate one specific pathogenic factor of clinical patients. On the other hand, most animal models are characterized by histological phenotypes and a few key molecular markers of TMJOA. Many models mimic the phenotype, but the similarity to the underlying molecular components of human TMJOA is typically not known.

As mentioned earlier, rodents are the most frequently used animals for TMJOA modeling. The primary disadvantages of these models are related to differences in anatomical structure and joint mechanics between these species and humans. The mandibular condyles of rodents extend antero-posteriorly, whereas in humans, the direction is lateromedial (Bermejo et al., 1993). The anterior-posterior axial length of the condyle is about 5 mm in rats and about 10 mm in rabbits, both of which were much smaller than that in humans (Orset et al., 2014; Monteiro et al., 2021). Moreover, the condyle axis is sagittal in rodents for propulsion movement, whereas it is transversal in humans for tridimensional motions, including opening, deduction, and propulsion (Orset et al., 2014). Some differences in disease expression between animal and human remain inexplicable. Unlike in humans, the incidence and severity of TMJOA is higher in male mice than in female mice (Silberberg and Silberberg, 1963). Even so, the advantages of small animal models include relatively low cost, ease of handling, more rapid disease progression, and availability of housing. As a result, they have been particularly popular for evaluating new therapeutic interventions and investigating the pathological process of TMJOA.

The advantage of large animal models is that they are anatomically similar to humans, particularly in joint size and cartilage thickness. Pigs have been regarded as the most suitable experimental model for human TMJ due to their similar condyles, articular disc, and mechanical properties to those of humans (Herring et al., 2002; Sun et al., 2002). However, disadvantages of large animal models are primarily related to the high costs, long maturation periods, and slow disease progression. Due to the rapid progress of TMJOA in small animals, the TMJ in small animals can be used for screening of potential therapeutics. The efficacy of drugs in small animals may not accurately reflect the efficacy observed in human TMJOA. Therefore, the TMJs from large animals, such as pigs and sheep, are still needed for preclinical studies to evaluate the clinical processes and their treatment in TMJOA. Despite various problems, animal models are still irreplaceable at least in the study of the pathology and progression of TMJOA rather than the etiology of TMJOA.

## Discussion

Animal models of TMJOA are important tools for studying the pathogenesis of TMJOA and evaluating potential therapeutic interventions. The value of these models mainly depends on how well they correspond with human disease. Various methods are used to build disease models of TMJOA, but each kind of model has its limitations. For each new study, considering the application of each model may help guide model selection. Different animal models could induce different TMJOA lesions. For example, chemical models can be used for the study of pain mechanisms; surgical models may be optimal for therapeutic study. Mechanical models may be appropriate for the study of pathogenesis; naturally occurring models would provide best models for studying aging phenotype; and genetically modified models are required for research of specific genes.

Animal models of OA can be classified into five categories: naturally occurring, genetically modified, surgically induced, chemically induced, and non-invasive animal models (Lampropoulou-Adamidou et al., 2014; McCoy, 2015). Some laboratory animals, such as certain strains of Syrian hamsters, dogs and cynomolgus macaques can develop OA spontaneously. These animals have not been used in studies of TMJOA yet. Since the anatomical structure and physiological composition of other synovial joints are different from those of the TMJ, most surgically induced models and non-invasive models cannot be applied to the modeling of TMJOA. Among them, impact loading has been applied to TMJ and successfully induced OA-like lesions in TMJ (Wang et al., 2008). In addition to the five drugs used for intra-articular injection models, quinolone and carrageenan are commonly used in other synovial joints to induce OA (McCoy, 2015). These drugs can be used to induce TMJOA in the future.

Age is one of the strongest risk factors for OA. The incidence and severity of TMJOA increase with age (Haskin et al., 1995). In contrast, most preclinical studies are conducted in young animals. For example, in the most widely used rat model of TMJOA, namely unilateral anterior crossbite (UAC), mechanical loading is usually performed on 6-8-week-old rats. However, comparison of 6-week-old and 28-week-old rats revealed that age affects the basal pattern of gene expression in joint tissues. When UAC is performed on 28-week-old rats, the ensuing TMJOA is more severe than in young rats (Zhang Y. et al., 2021). Since OA-like lesions occurr spontaneously with age in naturally occurring models, various induction methods can be used on these animals to study how other stimulus and aging synergistically affect TMJ.

Reports on the prevalence of TMJOA have shown significant gender differences (Zhao et al., 2011; Li et al., 2020). Its preponderance in women and early onset during reproductive years according to epidemiological research are totally different from the epidemiological characteristics of other joints, such as in knee OA, which primarily happens to postmenopausal women (Zhao et al., 2019; Dai et al., 2020). Severe TMJOA has been reported in young females whose blood oestrogen levels were medically low (Gunson et al., 2009), thus the relationship between TMJOA and oestrogen has attracted much attention. Studies have shown that oestrogen has an important effect on bone and cartilage metabolism. Oestrogen can regulate the secretion of cytokines and affect some key metabolic pathways to regulate bone and cartilage metabolism (Wang Q. P. et al., 2012). Several animal studies have confirmed that oestrogen deficiency leads to cartilage degeneration in the condyle, and results in more severe TMJOA-like lesions in the presence of mechanical stress stimulation (Nogami et al., 2020; Zhang J. et al., 2021). More studies are needed to further explore the role of oestrogen in the pathogenesis of TMJOA and related molecular mechanisms.

Notably, TMJOA involves not only cartilage but all the joint tissues; therefore, analysis of cartilage and periarticular tissues is recommended *in vivo* studies. Most of the studies evaluated cartilage degeneration and bone reconstruction by histology, histomorphometry, and immunohistochemistry. Only few studies analyzed the changes in articular disc, synovial membrane, and temporal surface. Therefore, the pathological changes in the whole joint should be studied to understand the etiological mechanism of TMJOA from a comprehensive perspective.

Over the past 40 years, TMJOA animal models have undoubtedly improved our understanding of the pathophysiology of the disease and contributed to the development of disease-modifying therapies. This review presents an overview of animal models used to study TMJOA, as well as the usefulness, histopathological changes, and scope of application of each model and current animals used in TMJOA research. Although many methods are used to build disease models, no single ideal animal model has been established for the comprehensive study TMJOA. This is because current models are mostly single-factor models, which cannot fully reflect the etiology and progression of TMJOA. In the future, the modeling approach should be improved, and more multi-factor models should be established to provide more suitable animal models for further study of TMJOA.
